# Metabolic reprogramming in the tumor microenvironment: unleashing T cell stemness for enhanced cancer immunotherapy

**DOI:** 10.3389/fphar.2023.1327717

**Published:** 2023-12-19

**Authors:** Youhan Liu, Tao Wang, Wen Ma, Zixuan Jia, Qinglu Wang, Maoling Zhang, Ying Luo, Hongmei Sun

**Affiliations:** ^1^ College of Sport and Health, Shandong Sport University, Jinan, China; ^2^ Department of Pediatric Surgery, Zibo Central Hospital, Zibo, China; ^3^ Department of Clinical Laboratory, Zibo Central Hospital, Zibo, China

**Keywords:** tumor microenvironment, H^+^, lactate, K^+^, cancer

## Abstract

T cells play a pivotal role in the immune system by distinguishing between various harmful pathogens and cancerous cells within the human body and initiating an immune response. Within the tumor microenvironment (TME), immune effector T cells encounter both immunosuppressive cells and factors that hinder their functionality. Additionally, they endure robust and persistent antigenic stimulation, often leading to exhaustion and apoptosis. However, the stemness of T cells, characterized by their ability to survive and self-renew over extended periods, represents a primary target in immune checkpoint therapies such as anti-PD-1 therapy. T cell stemness encompasses specific memory T cell subsets and progenitor-exhausted T cells with stem cell-like properties. Therefore, understanding the impact of the TME on T cell stemness, including factors like K^+^, lactate, and H^+^, holds significant importance and can facilitate the mitigation of terminal T-cell depletion, the identification of potential resilient biomarkers or therapeutic targets resistant to immune checkpoint therapies, and ultimately lead to sustained anti-tumor effects. Thus, it offers a novel perspective for advancing tumor immunotherapy.

## Introduction

Malignant tumors represent a significant global health challenge, characterized by elevated incidence and mortality rates, posing a severe threat to human well-being. In 2019, the World Health Organization reported that cancer stands as the second leading cause of death among individuals under the age of 70 in 112 of 183 countries worldwide (Ferlay et al., 2021). Recent investigations have revealed a strong association between the emergence and progression of tumors and the tumor microenvironment (TME) (Xiao and Yu, 2021), and alterations in the TME exert profound effects not only on the immune response within tumors but also on the efficacy of tumor treatments (de Visser and Joyce, 2023).

In addition to conventional approaches such as surgery, chemotherapy, and radiotherapy, the field of malignant tumor treatment has witnessed rapid advancements in research areas such as stem cell biology, immunology, molecular technology, and tissue engineering. Immunotherapy, recognized as a safe and effective treatment modality, has found an increasingly prominent role in comprehensive cancer management (Lu et al., 2022). Among the immune cells operating in the TME, T lymphocytes, especially CD8^+^ T cells, have been identified as pivotal players in the eradication of cancer cells (Reiser and Banerjee, 2016). Nonetheless, many cancer treatments are limited by T cell exhaustion (Yang et al., 2022), and addressing the causes of T cell exhaustion to sustain their stemness has been shown to enhance the efficacy of immunotherapy in tumor treatment, resulting in superior anti-tumor capabilities (Vodnala et al., 2019; Li et al., 2020; Mo et al., 2021). T cell stemness, characterized by the potential for prolonged survival, self-renewal and immune reconstitution, has garnered substantial evidence supporting its therapeutic relevance (Gattinoni et al., 2017; Morrot, 2017). Immunotherapy, which can harness the inherent capacity of T cells, is now considered a highly effective option for the treatment of cancer patients. Herein, this article focuses on investigating the impact of alterations in the TME on T cell stemness.

T cell stemness has been observed to be influenced by a range of factors, including the androgen receptor (Yang et al., 2022), G protein signaling 16 (Weisshaar et al., 2022), and changes in the TME (Vodnala et al., 2019; Feng et al., 2022; Xie et al., 2023). The TME represents a complex, interconnected system comprising components within and surrounding tumor cells. Beyond the tumor cells themselves, the TME can be broadly categorized into the immune microenvironment, primarily composed of immune cells, and the non-immune microenvironment, primarily comprised of fibroblasts. Additionally, ions and microorganisms are also present within the TME (Smetana and Masarik, 2022; de Visser and Joyce, 2023). Notably, a distinctive hallmark of the TME is hypoxia, which compels tumor cells to rely on anaerobic glycolysis for energy metabolism, resulting in lactate accumulation (Vaupel and Multhoff, 2017; Wei et al., 2021). Furthermore, ion-exchange proteins on the tumor cell membrane continuously transport intracellular H^+^ ions to the extracellular space (Chen and Pagel, 2015; Wei et al., 2021). Moreover, studies have revealed that both in murine and human tumor cells, cell necrosis releases intracellular K^+^ ions into the extracellular fluid, elevating K^+^ ion concentrations in the TME (Eil et al., 2016). Elevated levels of K^+^, lactate, and H^+^ in the TME have the potential to diminish T cell apoptosis, thereby promoting T cell stemness (Vodnala et al., 2019; Feng et al., 2022; Cheng et al., 2023). Given that T cell stemness can have long-lasting anti-tumor effects in immunotherapy, in this brief review, we summarize the effects of TME changes on T cell stem-cell properties for providing insights into optimizing and enhancing immunotherapy strategies.

## T cell status

T cells are highly heterogeneous and can be categorized into distinct subsets based on their activation status, including naive T cells, effector T cells, and memory T cells (Lanzavecchia and Sallusto, 2005). Upon activation, naive T cells differentiate into effector T cells, which do not engage in lymphocyte recirculation but rather migrate to peripheral inflammatory sites or specific organ tissues to execute immune responses, often culminating in exhaustion (Wherry and Kurachi, 2015). On the other hand, another portion of naive T cells differentiate into memory T cells, which represent two key states: T cell stemness and exhaustion. T cell stemness describes the stem cell-like behavior of T cells, including self-renewal, multipotency, and sustained functionality (Vodnala et al., 2019; Li et al., 2020). On the other hand, T cell exhaustion signifies the progressive impairment of effector functions due to prolonged to antigens, and exhausted T cells (T_EX_) exhibit high expression of various inhibitory receptors and demonstrate severe defects in cell proliferation and cytokine production (Wherry and Kurachi, 2015; Gonzalez et al., 2021). Therefore, it can be confirmed that the TME harbors diverse T cell states including naive T cells, effector T cells, T_EX_, senescent T cells, and stem-like T cells, each assuming distinct roles (Crespo et al., 2013). Mouse studies have demonstrated that blocking T cell differentiation can stimulate the generation of T cell stemness (Crespo et al., 2013).

## T cell exhausted

T cell exhaustion signifies the gradual decline of inflammatory and antigen-responsive T cells during chronic infections and cancer progression. They progressively lose their effector functions and memory T cell characteristics, leading to an inability of the body to sustain a durable and effective immune response (Wherry, 2011), and in this state, the T cells become resistant to restimulation (Crespo et al., 2013). T cell exhaustion is mainly characterized by the loss of interleukin-2 (IL-2) production, diminished proliferative potential, reduced cytolytic activity, and decreased expression of interferon-γ (IFN-γ) and tumor necrosis factor-α (TNF-α), ultimately compromising their protective capabilities (Wherry, 2011). However, exhausted T cell exhibits heterogeneity in both phenotype and function and can be divided into two main subsets: progenitor-exhausted T (T_PEX_) cells and terminally T_EX_. T_PEX_ cells possess a stem-like exhausted phenotype characterized by the expression of PD-1, CD127, the chemokine receptor CXCR5, and high levels of *TCF-1* (encoded by *Tcf7*) (McLane et al., 2019). The *TCF-1* subset is pivotal for memory formation, as it possesses self-renewal and proliferative capacities and exhibits a favorable response to immunotherapy (Miller et al., 2019). *TCF-1* serves as a critical marker for T cell stemness (Wen et al., 2021). Compared to T_PEX_ cells, terminally T_EX_ display impaired proliferative potential and lack *TCF-1* expression, rendering them unresponsive to activation (Hudson et al., 2019; Siddiqui et al., 2019). Additionally, these two types of T_EX_ exhibit distinct metabolic characteristics. T_PEX_ cells predominantly engage in catabolic metabolism, characterized by mitochondrial fatty acid oxidation (FAO) and oxidative phosphorylation (OXPHOS) (Adams et al., 2016). On the other hand, terminally T_EX_ primarily relies on glycolytic metabolism. However, prolonged antigen stimulation can induce changes in mitochondrial structure, resulting in impaired glycolysis and OXPHOS (Scharping et al., 2016; Schurich et al., 2016). Notably, an immunotherapy trial in lymphoma patients revealed higher levels of T_PEX_ cells in lymph nodes without cancer metastases (Rahim et al., 2023).

## T cell stemness

The concept of T cell stemness was first recognized in 2001 when mouse central memory T cells (T_CM_) were experimentally inhibited at the pre-differentiation stage using transcriptional inhibitors, which resulted in the preservation of their replicative potential and their ability to generate effector T cells over the long term upon encountering a subsequent antigenic challenge (Fearon et al., 2001; Crespo et al., 2013). Recent investigations have revealed that T cells undergo a programmed sequential process involving activation upon stimulation and differentiation into stem cell-like cells (Gattinoni et al., 2009; Vodnala et al., 2019). Following stimulation of the T cell receptor (TCR), naive T cells become activated and can differentiate into effector T cells and memory T cells. Memory T cells can be further categorized into effector memory T cells (T_EM_), central memory T cells (T_CM_), tissue-resident memory T cells, and stem cell memory T cells (T_SCM_) (Pace, 2021). The differentiation of naive T cells into memory and effector states is primarily influenced by the intensity of the signals they receive, predominantly through the TCR (Vodnala et al., 2019). Studies have indicated that once effector T cells enter the TME, they are not only subjected to continuous antigen stimulation but also to immunosuppression and other limiting factors, which often leads to apoptosis after antigen clearance, with only a small fraction of T cells (5%–10%) surviving. These survivors are referred to as memory precursor T cells or progenitor-depleted T cells, and they subsequently differentiate into various subtypes of memory T cells. Consequently, reducing or altering the immunosuppressive conditions in the TME is crucial for generating T cells with stemness-like properties capable of long-term survival under sustained antigen stimulation, ultimately achieving an effective and enduring anti-tumor response (Wang et al., 2018; Li et al., 2022). In particular, T_SCM_ can maintain self-renewal and memory capacity for up to 25 years (Gao et al., 2021; Li et al., 2021; Fazeli et al., 2023). The differing lifespans of T cells in various states can be attributed to changes in their transcription factor promoters, with methylation imparting self-renewal ability while demethylation does not. Furthermore, T_SCM_ possesses telomeres that are protected from damage (Gao et al., 2021; Wang et al., 2021; Fazeli et al., 2023). T cells exhibiting stemness characteristics display high expression of stemness-associated genes, including *TCF-1*, *Il7r*, and *Cxcr3*, while showing low expression of the genes *Ifng*, *Gzmb*, *Gzmc*, and *Gzmf* (Feng et al., 2022). Additionally, they exhibit elevated expression of CD62L and CCR7, diminished expression of CD44, and the capacity to rapidly acquire effector functions like memory cells following TCR stimulation (Gattinoni et al., 2011).

Despite T cells with stemness characteristics exhibiting lower secretion of IFN-γ, TNF-α, and granzyme B (GZMB), their capacity for tumor cell destruction is diminished (Gattinoni et al., 2011). Nevertheless, research has demonstrated that these T cells with stemness properties possess enhanced self-renewal, sustained anti-tumor effect and proliferative potential, along with the ability to differentiate into various subtypes of memory cells (Gattinoni et al., 2011; Fuertes Marraco et al., 2015). Compared to naive T cells and effector T cells, stemness in T cells is associated with increased mitochondrial mass and a predominant reliance on FAO and mitochondrial OXPHOS for prolonged survival (Fox et al., 2005). Given their enduring persistence, T cells with stemness properties can be harnessed to enhance T cell-based immunotherapy against tumors. Studies have indicated that T cells with stemness attributes, as well as other memory-like T cell subsets like T_PEX_, exhibit longer-lasting anti-infection and anti-tumor functions, which prove advantageous for tumor immunotherapy (Golubovskaya and Wu, 2016; Vodnala et al., 2019; Biasco et al., 2021). Environmental factors, particularly those within the TME, exert a pivotal influence on the status of T cells. The TME may either enhance or suppress T cell function, making it a potential target for intervention. Therefore, understanding the factors within the TME that promote T cell stemness introduces a fresh perspective for advancing tumor immunotherapy.

## Tumor microenvironment high K^+^

### High K^+^ induces autophagy in T cells

Autophagy, first discovered in 1962, refers to the cellular process where autophagosomes are formed by cells to degrade organelles, proteins, and other components under the control of specific genes. Ultimately lysosomes are used to break down their own organelles and contents, facilitating cellular metabolism and renewal (Kundu and Thompson, 2008). Autophagy serves to prevent cellular damage and promote cell survival in response to various stressors, such as nutrient deprivation or hypoxia (Lum et al., 2005). One notable characteristic of the TME is the rapid division of cancer cells, which compete for limited local resources (Sitkovsky and Lukashev, 2005). These factors can result in cellular apoptosis and necrosis (Richards et al., 2011). However, cell death can also alter the extracellular environment and lead to the release of intracellular ions. Research has indicated that the concentration of K^+^ in the interstitial fluid of tumors is higher than that in normal tissue interstitial fluid (Zimmerli and Gallin, 1988). Elevated K^+^ levels significantly reduce glycolytic intermediates and essential amino acids in T cells, affecting nutrient uptake—a phenomenon termed “caloric restriction” by (Vodnala et al., 2019). Nonetheless, this reduction in nutrient availability does not diminish T cell viability or proliferative potential; rather, it induces autophagy in T cells (Vodnala et al., 2019). Autophagy occurs concurrently with an increase in the methyl donor S-adenosylmethionine (SAM) (DeVorkin et al., 2019). It was found that the increased deposition of *H3K27me3* in the high K^+^ situation of TME suggest a shift towards T cell stemness (Vodnala et al., 2019). The Kennedy pathway is the responsible for synthesizing phosphatidylethanolamine (PE), an early initiator of autophagosome formation and autophagy (Gibellini and Smith, 2010). The autophagy marker protein microtubule-associated protein LC3b-I is converted to LC3b-II through conjugation with PE. LC3b-II is a structural protein of autophagosomes, and its expression reflects the level of autophagic activity (Panda and Singh, 2023). Immunoblot analysis conducted under high K^+^ conditions confirmed an increase in LC3b-II, demonstrating that despite caloric restriction induced by T cells in a high K^+^ environment, mitochondrial integrity and oxygen-consuming capacity are preserved, which limits sustained energy consumption under high K^+^ conditions. Moreover, a decrease in the phosphorylation level of phosphorylation 3-kinase (PI3K)/AKT/mammalian target of the rapamycin (mTOR) signaling induces nutrient protection and a metabolic state resembling functional starvation, thereby prompting autophagy (Vodnala et al., 2019).

### High K^+^ on T cell activation pathways

Previous studies have demonstrated that PI3K-Akt-mTOR signaling promotes the differentiation of CD8^+^ T cells into effector T cells while concurrently inhibiting the generation of memory T cells (Rao et al., 2010; van der Waart et al., 2014; Crompton et al., 2015; Li et al., 2020). Elevated K^+^ in the TME also affects the activation process of T cells, which inhibits the functionality of TCR-driven effector proteins by suppressing the PI3K-Akt-mTOR pathway. Sustained strong TCR signaling may induce T cells to become terminal T cells and may predispose them to apoptosis. In contrast, weak TCR signals may not be sufficient to effectively activate T cells but may prevent T cell differentiation during immune initiation and arrest at the T_SCM_ stage (Wang et al., 2018). Thus, T cell activation is reduced, and the inhibition of the TCR by high K^+^ occurs mainly by reducing TCR activation-induced phosphorylation of AKT-targeted serine/threonine residues (Vodnala et al., 2019), via a mechanism involving serine/threonine phosphatases to regulate the activity of Akt downstream of PI3K (Eil et al., 2016; Vodnala et al., 2019). During increased levels of K^+^, the use of okadaic acid (OA), an inhibitor of the serine/threonine phosphatase PP2A21, was found to significantly restore T cell functions, and related analyses revealed that OA could reverse the reduced phosphorylation of Akt and S6 induced by elevated K^+^. Furthermore, disruption of the PP2A gene also reversed T cell functionality, and high K^+^ inhibits TCR-induced PI3K-Akt-mTOR phosphorylation by depending on PP2A (Eil et al., 2016). Therefore, T cells cannot be activated, thus leading to their stemness.

### High K^+^ leads to T cell stemness

Elevated levels of K^+^ in the TME initiate functional metabolic restrictions in T cells, prompting a starvation response and modifying cellular metabolism. Under high K^+^ conditions, it has been observed that there is an increase in the total acetyl coenzyme A (AcCoA), but the levels of AcCoA and its precursor citrate decrease in the cell nucleus (Pietrocola et al., 2015). This alteration subsequently affects gene expression (Eil et al., 2016; Vodnala et al., 2019). High K^+^ primarily maintains T cells in a stem-like state by influencing *TCF-1* expression (Vodnala et al., 2019). In human T lymphocytes, K^+^ efflux is regulated by two K^+^ channels: Kv1.3 (a voltage-gated K^+^ channel activated by membrane depolarization) and KCa3.1 (a K^+^ channel activated by increased cytoplasmic Ca^2+^; also known as IK1 or Gardos channel) (Srivastava et al., 2009). Additionally, it has been observed that blocking Kv1.3 and KCa3.1 channels in the TME with high K^+^ levels can inhibit T cell function, driving T cells toward a stemness state (Feske et al., 2015).

Kir2.1, an inward rectifying potassium channel, plays a significant role in this context. Knockout of the Kir2.1 channel in tumor-associated macrophages (TAMs) results in elevated K^+^ levels and induces a metabolic shift from OXPHOS to glycolysis (Chen et al., 2022). The reduction in nuclear-cytoplasmic AcCoA levels and its precursor citrate in T cells, along with the depletion of glycolytic metabolites, indicates a preference for AcCoA production and utilization in mitochondria for OXPHOS, aligning with the findings in TAMs (Vodnala et al., 2019). Thus, in conditions of elevated K^+^, the regulation of AcCoA influences histone acetylation and gene expression and initiates metabolic reprogramming. Studies have demonstrated alterations in the expression of acetyl-CoA synthetase 1 (Acss1), primarily located in mitochondria, under these conditions. These changes lead to metabolic reprogramming, enhanced oxidative capacity, and increased stem cell-like properties in T cells, suggesting that the Acss1 pathway may serve as a potential target for enhancing T cell stem cell-like capabilities (Vodnala et al., 2019).

Moreover, high K^+^ levels have been shown to inhibit the production of key cytokines such as IFN-γ, GZMB, and IL-2 in T cells (Vodnala et al., 2019). In CD8^+^ T cells exposed to high K^+^, an increase in the expression of the lymphoid homing marker CD62L and the co-stimulatory marker CD27 has been observed. Both *Tcf7* transcripts and proteins also exhibit an increase under high K^+^ conditions (Vodnala et al., 2019). Amino acids, including methionine, become depleted in T cells when exposed to elevated K^+^. Consequently, the metabolites required to support relevant aspects of T cell stemness are depleted (Vodnala et al., 2019). However, T cells subjected to high K^+^ conditions possess an enhanced capacity for recalling their response to antigens, remain relatively undifferentiated, and demonstrate greater persistence and self-renewal upon re-exposure to antigens (Gattinoni et al., 2012). T cells that regain their stemness can promptly acquire effector functions following stimulation via the TCR (Gattinoni et al., 2011).

## Tumor microenvironment high lactic

### Lactic on T cell function

Tumor cells undergo metabolic changes characterized by increased glucose uptake and a higher proportion of pyruvate conversion to lactic acid, even in normoxic conditions, and this phenomenon, known as aerobic glycolysis, is commonly referred to as the Warburg effect (Warburg, 1961). Previous studies have suggested that lactate plays a contributing role in tumor development and is detrimental to tumor therapy, There exists a correlation between lactate concentration in tumor tissue and the incidence of metastasis, with higher lactate concentrations associated with shorter survival (Walenta et al., 1997; Walenta et al., 2000; Walenta et al., 2004). Lactate within tumors fuels regulatory T cells and within progression of macrophages toward an M2 phenotype (Ohashi et al., 2017; Watson et al., 2021). Moreover, the rapid proliferation and growth of tumors can result in hypoxic conditions (Petrova et al., 2018), leading to energy metabolism through anaerobic glycolysis and the accumulation of lactic acid. Interventions targeting lactic acid can induce up to 60% cell death in cytotoxic T lymphocytes (CTLs) (Fischer et al., 2007). Lactic acid treatment leads to a decrease in the production of IFN-γ and IL-2 by CTL (Fischer et al., 2007), as well as reduced content of granule enzymes and perforin in T cells and NK cells (Fischer et al., 2007; Husain et al., 2013). The ability of lactic acid-treated CTLs to eliminate target cells is reduced by half compared to the control group. Elevated expression of monocarboxylate transporter 1 (MCT-1) in CTLs from melanoma patients suggests that lactic acid in the tumor environment hinders T cell function (Fischer et al., 2007).

However, T cells exposed to lactic acid exhibit a substantial increase in the expression of genes related to T cell functional and signaling, such as *Gzmb*, *Ifng,* and *TCF-1*, leading to the initiation of changes associated with T cell stemness, ultimately enhancing the long-term anti-tumor immune response in T cells (Feng et al., 2022). Notably, the administration of a sodium lactate solution was found to significantly decrease tumor growth when injected into tumor-bearing mice. Moreover, when sodium lactate was co-administered with either a PD-1 antibody or a tumor vaccine, synergistic anti-tumor effects were achieved (Feng et al., 2022). Thus, while lactate does hinder T cell function, it is plausible that T cells with stem-like characteristics possess enduring anti-tumor capabilities and contribute to the synergy in immune therapy. However, upon the *in vivo* removal of CD8^+^ T cells, the anti-tumor effect resulting from lactate intervention diminishes (Feng et al., 2022). Consequently, the anti-tumor efficacy of sodium lactate is mediated through T cells and is an outcome of T cell stemness.

### High lactic leads to T cell stemness

Lactic acid treatment was observed to significantly enhance the therapeutic effectiveness of anti-PD-1 treatment across various tumor models through reduced tumor growth rates and extended survival periods. *In vitro* intervention with lactic acid in T cells demonstrated a notable elevation in the expression of the stemness marker gene *TCF-1* (Feng et al., 2022). The primary mechanism through which elevated lactate affects T cell stemness predominantly involves the inhibition of histone deacetylase activity in CD8^+^ T cells, which leads to the retention of histone acetylation at the *TCF-1* locus during the differentiation of CD8^+^ T cells, with a notable 3.5-fold increase in acetylation levels at the *H3K27* locus, thereby resulting in a reduction in the apoptosis and depletion of CD8^+^ T cells (Feng et al., 2022).

## Tumor microenvironment high H^+^

### High H^+^ on T cell metabolism

During glycolysis, tumor cells continuously transport intracellular H^+^ ions to the extracellular space through ion exchange proteins on their cell membranes, preventing self-acidosis (Chen and Pagel, 2015; Wei et al., 2021). Acidosis is t a defining feature of solid tumors, characterized by a pH range of 5.7–7 (Ashby, 1966; Vaupel et al., 1989). Extracellular acidosis has the effect of enhancing the endocytosis of dendritic cells (DCs) and influencing macrophage activity, leading to increased expression of MHC class II and CD86 (Vermeulen et al., 2004; Colegio et al., 2014). Under prolonged exposure to high extracellular H^+^ conditions, T cells experience reduced glycolysis and amino acid metabolism. Conversely, short-term exposure to high H^+^ levels only inhibits glycolysis without significant effects on amino acids (Cheng et al., 2023). High H^+^ levels severely impede glucose entry into the tricarboxylic acid cycle in T cells while promoting the breakdown of palmitic acid into AcCoA and citrate, resulting in metabolic reprogramming. Consequently, high H^+^ levels stimulate FAO in T cells (Cheng et al., 2023). T cells exhibit a higher oxygen consumption rate (OCR) and spare respiratory capacity (SRC) as characteristic trait of long-lived memory T cells (van der Windt et al., 2012; Cheng et al., 2023). This metabolic reprogramming mainly induces inhibition of the PI3K -AKT-mTOR signaling pathway, thereby inhibiting TCR activation and favoring the formation of CD8^+^ T cells stemness (Pollizzi et al., 2015; Ron-Harel et al., 2016; Cheng et al., 2023).

### High H^+^ triggers metabolic reprogramming of T cells

There is increasing evidence indicating that one-carbon metabolism plays a crucial role in determining cellular fate and influencing their functional states (Xie et al., 2023). Effector T cells characterized by stemness exhibit heightened glycolytic activity and rely on one-carbon metabolism. Conversely, T cell stemness is distinguished by unique metabolic traits, including increased FAO and SRC, which are critical for sustaining effective anti-tumor capabilities (Cheng et al., 2023). Therefore, metabolic reprogramming is crucial for the effective acquisition of stemness in T cells and their long-term survival. High H^+^ inhibits the expression of enzymes related to the methionine cycle (*MTR*, *AHCY*, and *BHMT*) as well as genes involved in folate metabolism (*SHMT1* and *SHMT2*) in T cells (Cheng et al., 2023). The expression of methionine transporter SLC7A5 on the surface of T cells is dependent on Myc gene (c-Myc) expression (Han et al., 2021). Elevated H^+^ concentrations can suppress c-Myc expression, further diminishing the expression of SLC7A5 and impeding the uptake of extracellular methionine by T cells, this ultimately hampers the production of SAM (Ron-Harel et al., 2016). As a methyl donor for histone methylation modifications, reduced levels of SAM can lead to alterations in histone modifications within T cells, thereby affecting gene expression. Investigations of various tumor-infiltrating CD8^+^ T cell subpopulations, revealed decreased expression of both c-Myc and SLC7A in T cell stemness.

### High H^+^ leads to T cell stemness

Under high H^+^ conditions (10 mM) *in vitro* culture significantly increased the expression of BACH2, CCR7, LEF1, and *TCF7* expression in CD8^+^ T cells. Additionally, research has demonstrated a H^+^ concentration-dependent effect on the expression of *CCR7* and *TCF-1* expression. Under high H^+^ conditions, both human and murine CD8^+^ T cells exhibit a substantial increase in *TCF-1* expression at the protein level, a key factor associated with stem properties. These T cell stemness also display heightened expression of *CCR7* and CD62L, along with a notable reduction in the production of IFN-γ and tumor necrosis TNF-α within the cells (Cheng et al., 2023). Quinn *et al* revealed demonstrated that exposure to a high-lactate, low-glucose environment induces reductive stress, ultimately resulting in decreased glycolysis and reduced serine production in effector and regulatory T cells (Quinn et al., 2020). High H^+^ levels lead to a decrease in inhibitory histone marks *H3K27* and *KLFe3* at memory-related gene loci (such as *TCF7*, *CCR7*, *ID3,* and *LEF2*) in T cells. Treatment with high H^+^ treatment significantly inhibits the expression of the methyltransferase EZH2, which governs *H3K27me3* modification, ultimately lowering intracellular levels of *H3K27me3* (Cheng et al., 2023). The decrease in *H3K27me3* induced by extracellular acid treatment promotes the transcriptional activation of stemness-related genes within T cells, ultimately maintaining the stem-like characteristics of T cells; therefore, T cells cultured under high H^+^ conditions exhibit strong anti-tumor activity *in vivo* (McLane et al., 2019).

## Conclusion

The adoptive transfer of tumor antigen-specific T cells has marked a significant advancement in cancer therapy, often resulting in the complete regression of certain malignant tumors, which depends largely on the persistence and differentiation status of the infused T cells. Preferably, less differentiated memory T cells with lower levels of differentiation are chosen for adoptive T cell transfer (ACT) due to their possession of stem cell-like attributes, including self-renewal and multipotent properties. Thus, T cell stemness plays a pivotal role in tumor immune surveillance and immunotherapy.

In recent years, there has been a growing interest in preclinical trials involving immune cells with stem cell characteristics for use in immunotherapy. The extended lifespan and robust proliferation potential make T cell stemness cells an ideal candidate for immunotherapy. Stem-like T cells can originate directly from naive lymphocytes, and notably, T_PEX_-phase cells are also regarded as stem-like T cells (Wen et al., 2021).

**FIGURE 1:**
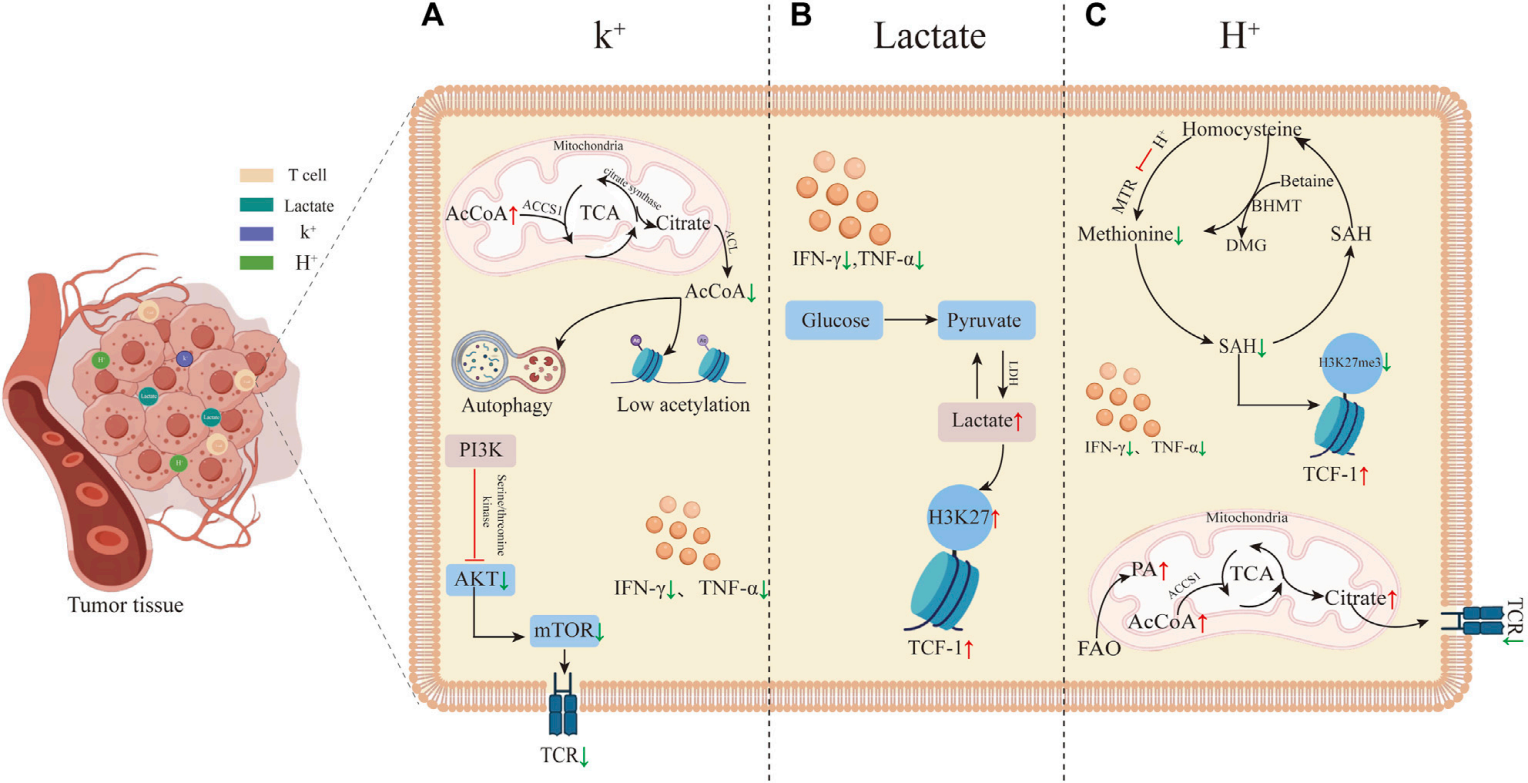
Tumor microenvironment-induced T cell stemness. **(A)** High K^+^ levels in the tumor microenvironment promote T cell stemness. High K^+^ leads to an increase in AcCoA within mitochondria while decreasing it in the nucleus, inducing autophagy and histone hypoacetylation. These alterations in gene expression reduce T cell differentiation into effector T cells by inhibiting the TCR signaling pathway. The inhibition of TCR by high K^+^ primarily occurs through reduced phosphorylation of Akt-targeted serine/threonine residues upon TCR activation. **(B)** Elevated lactate in the tumor microenvironment induces T cell stemness by increasing acetylation levels at the *H3K27* locus within the *TCF-1* gene during CD8^+^ T cell differentiation. This elevated acetylation preserves cellular stemness. **(C)** T cell stemness induced by high H^+^ levels in the tumor microenvironment. Elevated FAO results in increased AcCoA levels, leading to TCR inhibition, reduced T cell activation, and inhibition of methionine synthase MTR, thereby decreasing methionine synthesis. This reduction further lowers the synthesis of SAM and S-adenosylhomocysteine (SAH). SAM is a critical methyl donor for DNA and histone methylation reactions. The decreased methylation levels, particularly the histone marker *H3K27me3*, enhance *TCF-1* expression.

Recent studies uncovered that the TME exerts a notable influence on the development of T cell stemness. Despite prior findings indicating the suppressive role of lactate and acidic microenvironments in tumor immunity, it has been discovered that elevated levels of K^+^, lactate, and H^+^ all can restore and sustain T cell stemness by reshaping the metabolism within the T cells (Figure 1). T cell stemness not only enhances the efficacy of immunotherapy but also endures longer *in vivo*, leading to a more robust anti-tumor response (Bailey et al., 2017). The primary mechanism by which these three factors in the TME modify T cell stemness involves metabolic reprogramming, subsequently influencing gene expression, with a particular focus on altering *TCF-1* gene expression. *TCF-1* possesses intrinsic histone deacetylase (HDAC) activity, contributing to the maintenance of T cell stemness by repressing genes inappropriate for the T cell lineage (e.g., Cd4, Foxp3) (Xing et al., 2016). There is growing mounting evidence supporting the notion that metabolic pathways determine the fate of T cells and shape their epigenetic and functional states. Among the three treatment approaches aimed at regulating the metabolic reprogramming of T cells, the control of cellular energy intake or the targeting of molecules such as AcCoA represents a novel therapeutic strategy, to enhance resistance against T cell exhaustion. Furthermore, this paper introduces additional possibilities for improving tumor immunotherapy, suggesting that elevating K^+^ and lactate levels, along with increasing H^+^, can augment T cell stemness and, consequently, enhance the effectiveness of immunotherapy. Of immunotherapy.
